# Leukocyte Membrane Enzymes Play the Cell Adhesion Game

**DOI:** 10.3389/fimmu.2021.742292

**Published:** 2021-11-23

**Authors:** Georgina I. López-Cortés, Laura Díaz-Alvarez, Enrique Ortega

**Affiliations:** Department of Immunology, Instituto de Investigaciones Biomédicas, Universidad Nacional Autónoma de México, Mexico City, Mexico

**Keywords:** moonlighting protein, ectoenzyme, cell adhesion, signal transduction, leukocytes

## Abstract

For a long time, proteins with enzymatic activity have not been usually considered to carry out other functions different from catalyzing chemical reactions within or outside the cell. Nevertheless, in the last few years several reports have uncovered the participation of numerous enzymes in other processes, placing them in the category of moonlighting proteins. Some moonlighting enzymes have been shown to participate in complex processes such as cell adhesion. Cell adhesion plays a physiological role in multiple processes: it enables cells to establish close contact with one another, allowing communication; it is a key step during cell migration; it is also involved in tightly binding neighboring cells in tissues, etc. Importantly, cell adhesion is also of great importance in pathophysiological scenarios like migration and metastasis establishment of cancer cells. Cell adhesion is strictly regulated through numerous switches: proteins, glycoproteins and other components of the cell membrane. Recently, several cell membrane enzymes have been reported to participate in distinct steps of the cell adhesion process. Here, we review a variety of examples of membrane bound enzymes participating in adhesion of immune cells.

## Introduction

Enzymes play crucial roles in all life processes and its perpetuation because they facilitate most biochemical reactions, both within and outside the cells. Enzyme function is determined not only by substrate specificity and enzymatic properties, but, notably, by its cell compartmentalization. Ectoenzymes are membrane-bound enzymes which the catalytic site is found outside the cell, they mainly reach its position by means of vesicles or carrier proteins (1). Ectoenzymes are a widely heterogeneous class of enzymes essential to homeostasis maintenance. They regulate the concentration and activity of certain molecules in the extracellular milieu, such as hormones, nucleotides, bioactive peptides (2), etc. Several ectoenzymes have been reported to perform other functions aside of catalyzing chemical reactions (3), reason for which they have been included in the list of moonlighting proteins (http://www.moonlightingproteins.org/). A moonlighting protein has multiple functions that are not the result of gene fusion, distinct RNA splice variants, or proteolytic fragments. It has been postulated that moonlighting proteins originally had a unique function but have acquired others, in many cases by virtue of post-translational modifications. The origin and the enzymatic functions of moonlighting proteins are thoroughly discussed elsewhere (3, 4).

**Figure 1:**
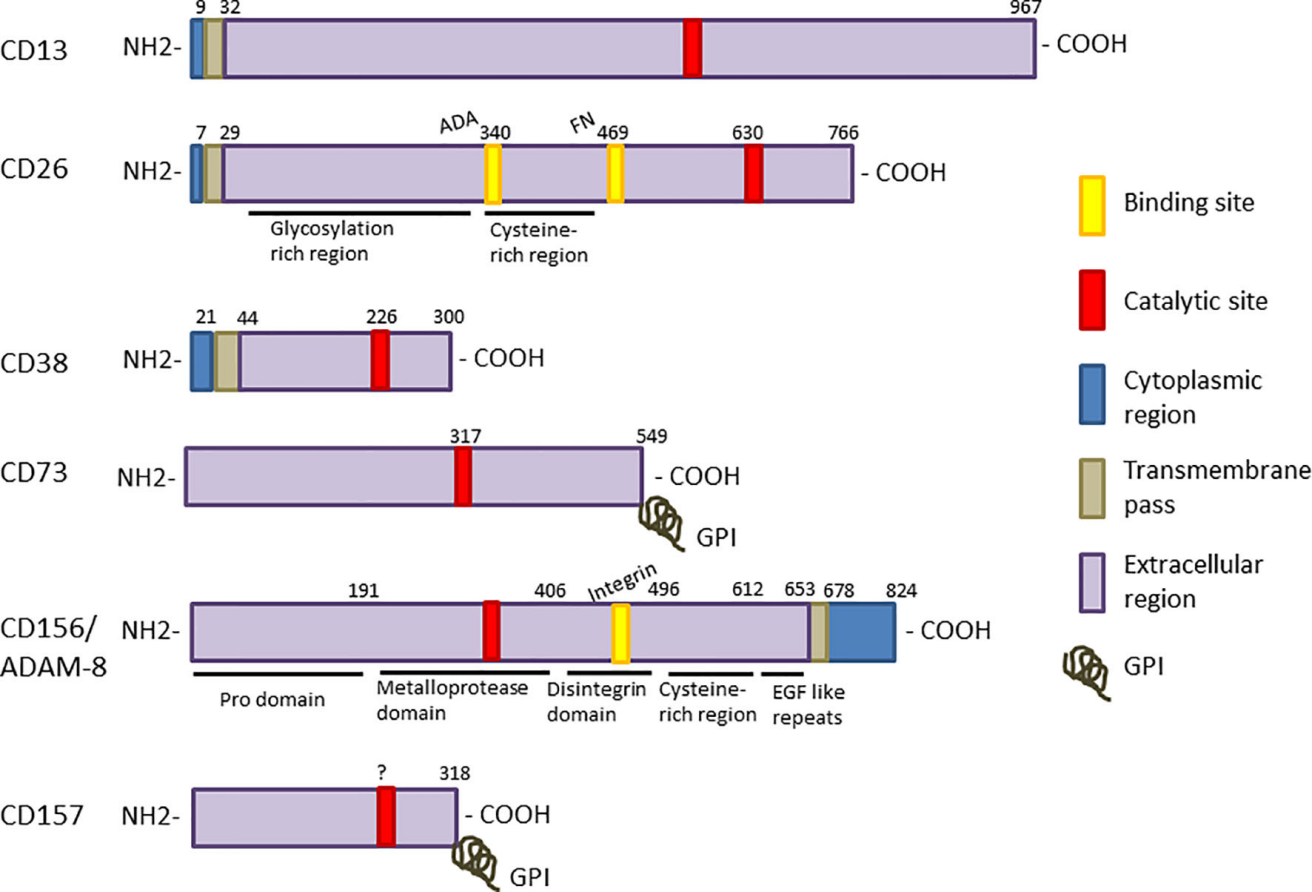
Linear representation each enzyme sequence. The different regions are displayed as color boxes: catalytic sites are red and the known binding sites for specific substrates, yellow. Intracellular regions are shown in blue, trans-membrane regions are represented as grey boxes, and extracellular regions are shown in purple. CD73 and CD157 are bound to the membrane through a glycosylphosphatidyl inositol molecule (GPI), symbolized by a grey coil. CD156/ADAM-8 is the only enzyme with signal transduction motifs (SH3 binding domains) in the cytoplasmic region.

Some moonlighting proteins are enzymes participating in cell adhesion processes (5–7). Cell adhesion enables cellular organization and communication in multicellular organisms. At the molecular level, it is a finely orchestrated process that includes the activation of canonical adhesion molecules, which trigger signal transduction cascades (8) and ultimately drive cell attachment to other cells or to the extracellular matrix (ECM). In leukocytes, cell adhesion is a critical process not only for cellular distribution in tissues, but also for establishing immunological synapses, mobilization, and migration. Such phenomena entail the coordination of several proteins. For example, leukocyte transendothelial migration requires molecules involved in protein-protein interactions that cause the deceleration of cells in circulation, proteins mediating the firm attachment of leukocytes to the endothelium and their squeezing through the endothelial junctions, and finally other proteins that mediate the active movement of cells through the tissues. Throughout these processes, membrane receptors initiate signal transduction pathways that regulate the cell’s adhesion properties and its changes in morphology. Therefore, it becomes evident that membrane adhesion molecules are activated at different time points to mediate either the approaching of cells’ membranes or strengthen their interaction, while in the intracellular compartment the cytoskeleton is rearranged (9). Here, we review the role of several ectoenzymes expressed by leukocytes involved in different steps of cell adhesion, and thus are considered as moonlighting proteins (**Table 1** and **Figure 1**).

**Table 1:** Expression and properties of the membrane enzymes that participate in cell adhesion in leukocytes.

Enzyme	Expression	Transmembrane pass	Cytoplasmic aa	Ligands	Interactions with other proteins on the same cell membrane	Associated to signal transduction
CD13	Endothelial cells, kidney and intestinal epithelial cells, monocytes, DC, macrophages, granulocytes, neurons	1	9	Unknown	FcγR I and II, possibly β1 integrin	Yes
CD26	Endothelial cells, intestine and lung epithelial cells, T and B lymphocytes and NK cells	1	6	ADA, Fibronectin, Collagen type 1, Caveolin-1	TCR, M6P/IGF-IIR, CXCR4, CD45	Yes
CD38	NK cells, T and B lymphocytes, HUVEC, thymocytes, monocytes, osteoclasts, platelets, erythrocytes, neurons, astrocytes, muscle cells, prostatic, pancreatic, kidney, retinal and corneal epithelial cells	1	21	CD31	TCR, BCR, CD19, CD81, class II MHC, CD16	Yes
CD73	Most B and T CD8+ lymphocytes, some CD4+ T cells, Th17, Treg, NK cells, follicular Dendritic Cells and myeloid derived suppressor cells, muscle cells, neurons, fibroblasts, reactive astrocytes, endothelial cells, and some epithelial cells.	Glycosyl phosphatidyl inositol anchored	None	Laminin, fibronectin, tenascin C, N-CAM, β2 integrin	Not determined	Yes
CD156	Monocytes, macrophages, granulocytes, dendritic cells, endothelial cells, B cells. In inflammatory conditions can be induced on osteoclast, lung epithelial cells, glial cells and neurons.	1	146 (has signaling motifs)	β1 integrin	β1 integrin	Yes
CD157	Neutrophils, monocytes, immature lymphocytes, bone marrow stromal cells, synoviocytes, endothelial and mesothelial cells, dermal fibroblasts	Glycosyl phosphatidyl inositol anchored	None	Fibronectin, type I Collagen, laminin, CD31	β1 and β2 integrins	Yes

## CD13

CD13, also Aminopeptidase N (ANPEP, gp150) (EC: 3.4.11.2) is a Zn^2+^-dependent metalloproteinase that catalyzes the cleavage of neutral amino acids at the N-terminal portion of peptides. CD13 is highly glycosylated and has a large extracellular region, a single transmembrane pass and a short cytoplasmic tail (10, 11). Through its enzymatic activity, CD13 modulates different processes, as it cleaves a wide variety of bioactive peptides, including Angiotensin III, Enkephalin, some cytokines, and others (10). CD13 is expressed by myeloid cells, endothelium, renal and intestinal epithelial cells, neurons and, importantly, it is overexpressed in various cancer types (12–15).

CD13 is particularly highly expressed in myeloid cells and leukemias, hence, it was used as a marker of myeloid cells even before it was known to be the same as the membrane enzyme Aminopeptidase N (16). CD13 expressed in myeloid cells has since been shown to participate in phagocytosis (17–19), cell adhesion (20, 21) and migration (22, 23). Evidence shows that CD13 participation in these processes is dependent on the activation of signal transduction cascades, and, at the same time, independent of the enzymatic activity, leading to the idea that CD13 could also be a receptor. Moreover, CD13 crosslinking with monoclonal antibodies triggers heterotypic and homotypic aggregation, i.e. between cells of different types or between cells of the same type, respectively. This is true for human neutrophils, monocytes and the monocytic cell line U-937 (21, 24). Cell aggregation induced by CD13 crosslinking with the anti-CD13 antibody mAb452 is dependent on signal transduction and independent of integrins (24). It is noteworthy that, while some anti-CD13 antibodies induce cellular aggregation (mAb 452, mAb MY7 and mAb WM15), others do not (mAb WM47) (21, 24), and there are two monoclonal antibodies (mAb E and mAb C) that disaggregate cell aggregates previously induced by mAb 452 (25). Little is known about the mechanisms involved, or about why different mAbs show different abilities to induce aggregation (24), but it is hypothesized that this could be related to the epitopes recognized by the mAbs.

Alongside well-studied adhesion molecules such as integrins and selectins, other molecules contribute or regulate transendothelial migration of leukocytes. Among them, CD13 participates by promoting monocyte adhesion to the endothelium (20), which is one of the initial stages of leukocyte exit from the blood vessels into the tissues. In a mouse model of peritoneal inflammation, the absence of CD13 reduced the amount of leukocyte infiltration, specifically of inflammatory monocytes, dendritic cells (DC) and neutrophils. The conclusion was that optimal monocyte infiltration is achieved when both monocytes and endothelial cells express CD13 (26).

Although there are reports identifying putative ligands (27), CD13 is still considered an orphan receptor because the natural ligand under physiological conditions remains undiscovered (10) and the short cytoplasmic tail opens the question of how it is able to transduce signals. On one hand, Riemann et al. observed, using Fluorescence Resonance Energy Transfer (FRET), that after crosslinking, CD13 co-localizes with FcγR I and II (CD64 and CD32, respectively) (28), suggesting that CD13 signaling activity stems from its association with molecules with canonical domains for signal transduction. On the other hand, it has been difficult to show the association of CD13 with other membrane proteins by immunoprecipitation. Nevertheless, it is clear that the signal transduction cascade involves CD13 phosphorylation in a Src-dependent manner (29), which causes the activation of the Focal Adhesion Kinase (FAK) and the approach of the scaffold protein IQGAP1, which mediates the remodeling of α-Actin (20, 22, 29, 30). Then, Ca^2+^ mobilization and PI3K activation ensue, leading to MAPK activation (31).

It was recently reported that CD13 participates in the recycling of β1 integrin (CD29). After the integrin activation by ECM-ligation, CD13 is phosphorylated and forms a complex with IQGAP1 and the small GTPase ARF6, which directs the β1 integrin to early endosomes (Rab5+) and then back to the membrane (22), this would result in an increase of the cell’s adhesive properties. Another report shows evidence that CD13 expression regulates the expression of Syndecan 1 (SNC1) and β4 integrin (CD104) by downregulating the activation of PKCδ (32), which would enhance adhesiveness. However, it is not clear whether CD13 directly modulates PKCδ activity, or it is an indirect effect. Additionally, it has been suggested that CD13 could also promote adhesion by interacting with the ECM, *via* molecules like Galectin-3 or fibronectin (33), or with other membrane molecules (24).

## CD26

The moonlighting protein CD26 is an enzyme of great relevance in T cell biology. CD26 participates in interactions with several proteins during T cell activation, establishing physical contact with the Antigen Presenting Cells (APC) so it reaches an accurate activation level, and enabling cell-cell communication. As an enzyme, CD26 [dipeptidyl peptidase IV (DPPIV) or Adenosine Deaminase Complexing protein 2 (ADCP2)] (EC: 3.4.14.5) is central to the regulation of multiple processes *via* its serine exopeptidase activity. It cleaves dipeptides from the N-terminus of peptides with proline or alanine at the penultimate position (34). Some of its substrates include Glucagon-like protein 1 (GLP-1) and the Gastric Inhibitory Protein (GIP) (35) (both important in glucose metabolism), as well as Substance P, and various chemokines. CD26 is a type II membrane protein with a short 6-aa cytoplasmic tail, a single transmembrane pass and a large 738-aa extracellular region that includes the catalytic site (35). The extracellular portion of CD26 contains 2 domains: a C-terminal serine protease domain homologous to α/β-hydrolases, and a propeller domain comprising two subdomains: a cysteine-rich region and a highly glycosylated region. CD26 forms homodimers on the cell membrane (36).

CD26 is expressed on the endothelium, epithelial cells of the kidney, liver, lung, intestine and on T cells, some B and NK cells, as well as on myeloid cells (37). In activated T cells, CD26 participates in lymphocyte adhesion to endothelial cells through interaction with another membrane enzyme, adenosine deaminase (ADA), expressed on endothelial cells (38). It was shown that CD26-ADA interaction increases lymphocyte adhesion to the endothelium, and it was suggested that the increased adhesion is mediated by the integrin lymphocyte function-associated antigen-1 (LFA-1, CD11a/CD18), because CD26-ADA interaction promotes the high affinity conformation of the integrin (38). The mechanism through which LFA-1 is activated is not clear, but it certainly involves an inside-out signaling pathway. ADA is implicated in the immunomodulation of adenosine, and both CD26 and ADA are expressed at higher levels on T effector cells than on Treg cells.

It has been suggested that CD26 regulates multiple T-cell processes, including maturation, migration, activation and cytokine secretion (39). Several proteins involved in T-cell activation interact with CD26 in an enzymatic activity-independent manner, including Mannose 6-Phosphate/Insulin-like Growth Factor II receptor (M6P/IGF IIR), CD45 (40), Caveolin- 1, Fibronectin (FN), Collagen type 1, Streptokinase, CXCR4, Plasminogen type 2, HIV gp120 protein, human coronavirus MERS-CoV Spike protein and the extracellular Adenosine Deaminase (ADA) (23, 39, 41–44). Moreno et al. demonstrated a physical interaction between CD26-bound ADA with Adenosine Receptor 2 (A2R), possibly through a molecular bridge between lymphocytes expressing CD26 and DCs expressing A2R (45). Also, CD26 on T cells is presumed to interact with Caveolin-1 on APCs, which could contribute to the overall interaction between these cells (40). In fact, ADA and Caveolin-1 bind to CD26’s highly glycosylated region while FN, Collagen type 1, Plasminogen and Streptokinase bind to the cysteine-rich region (35).

Multiple reports establish the ability of CD26 to directly bind to FN and Collagen type I in the ECM (46). Metastatic cancer cells and blood-born cancer cells move toward different tissues *via* interactions between CD26 and a polymeric form of FN expressed in lung epithelial cells (34). Similarly, Sato et al. demonstrated in a T-anaplastic large cell lymphoma cell line (T-ALCL), Karpas 299, that CD26 modulates the phosphorylation of β1 integrin by inducing the activation of p38 Mitogen Activated Protein Kinase (MAPK) (47). This suggests that CD26 mediates cell adhesion to the ECM through MAPK-dependent phosphorylation and β1 integrin activation.

CD26 is considered as a potential target for cancer therapy, because it is a marker of multiple types of cancer cells (44, 48, 49). In a model using mice inoculated with Karpas 299 lymphoma cells expressing or not CD26, CD26 was shown to be necessary for tumor development (47). As aforementioned, CD26 participates in cell adhesion to the ECM and the endothelium through its binding sites for FN, Collagen type I and ADA. The anti-CD26 antibody 6A3, which blocks the FN binding site on CD26, decreased adhesion of cancer cells (50).

## CD73

The enzyme ecto-5’ nucleotidase (Ecto-5’-NT) (EC: 3.1.3.5), CD73, or Lymphocyte-Vascular Adhesion Protein 2 (LVAP-2) is a glycosyl phosphatidyl inositol-anchored membrane protein involved in the metabolism of nucleotides. Ecto-5’-NT catalyzes the dephosphorylation of nucleotide monophosphates into their corresponding nucleosides (51, 52). It is expressed on the membrane as an homodimer, although it can be also found intracellularly and as a soluble form in circulation (52).

Minimal concentrations of nucleotides are released physiologically into the extracellular space as messengers (53), but injuries or mechanical damage may result in an increased release of nucleotides into the extracellular space, leading to an inflammatory response (54). Conversely, high concentrations of extracellular adenosine downregulate pain, inflammation, proliferation and cytokine secretion (53). Hence, CD73 has been proposed as a key regulator of inflammation and a prominent target in chronic pain (54, 55). CD73 and CD39 work together on the cell membrane regulating of the adenosine metabolism; for example, Treg cells and myeloid- derived suppressor cells (MDSCs) use the nucleotidase CD39 to cleave ATP into AMP, which then is dephosphorylated into adenosine by CD73 (56, 57). The nucleosides can subsequently be transported into the cell through purinergic receptors such as A2A (53), and once inside the cell, be phosphorylated again for different purposes, including cell proliferation (58, 59). The increased expression of CD73 and CD39 produce increased extracellular concentrations of adenosine, which contributes to the immunosuppressive microenvironment of tumors and it has been demonstrated that they both are regulated by the hypoxia- inducible factor- α (HIF-1α) (57). Thus, inhibition of the enzymatic activity of these enzymes has been proposed as a potential therapeutic strategy to combat cancer (60).

CD73 is widely expressed. In the hematopoietic lineage, it is present on mature B lymphocytes, some CD8^+^ T cells, Tregs, and Follicular Dendritic Cells (FDC) (61). Other non-hematopoietic cells also express CD73, such as fibroblasts, some epithelial and endothelial cells, skeletal muscle cells and neurons and, significantly, a variety of solid tumors (52, 62). In addition to its enzymatic activity, the role of CD73 in different immunological processes has also been studied, including lymphocyte activation, proliferation, cell adhesion, and the formation of germinal centers in secondary lymphoid organs (59, 63, 64).

As an adhesion molecule, CD73 participates in cell interactions with sulphated proteins of the ECM, such interactions potentially contribute to regulate invasiveness and metastasis of cancer cells (65). Chicken’s gizzard CD73 interacts with columns containing Laminin or Fibronectin bound to sepharose beads, which reduces its enzymatic activity (65–68). Human CD73 also interacts with the ECM protein Tenascin C, important for adhesion of MDA-MB-231 breast cancer cells; this interaction inhibits 75% of CD73’s enzymatic activity (69). These observations make it highly likely that these ligands bind near the catalytic site.

In addition to ECM proteins, there is evidence that CD73 interacts with CD56, Neural-Cell Adhesion Molecule (N-CAM) (64) and CD18, the β chain of the integrin Lymphocyte Function-Associated Antigen-1 (LFA-1, integrin αL/β2, CD11a/CD18), enabling cellular adhesion between endothelial cells and lymphocytes (70, 71). Moreover, CD73 is known to be chopped off upon crosslinking with mAbs on lymphocytes but not on endothelial cells, suggesting a mechanism for regulation of lymphocyte-endothelial cell adhesion (59). CD73 interaction with LFA-1 may be the major mechanism by which CD73 contributes to enhance cell adhesion of lymphocytes to endothelial cells and along with its enzymatic activity, it could represent a major function of this moonlighting protein (64).

## CD38

The ADP-ribosyl cyclase 1 or CD38 (EC: 2.4.99.20, EC: 3.2.2.6) is a conserved enzyme with 10 cysteines that are essential for maintaining its tertiary structure and for its catalytic activity (72). CD38 catalyzes the reaction of breaking up nicotinamide adenine dinucleotide (NAD^+^) into adenine dinucleotide phosphate ribose (ADPR), cyclic ADP ribose cADPR and nicotinamide (73–75). cADPR is a second messenger that induces Ca^2+^ mobilization independently from 3,4,5-inositol triphosphate (IP3) (74, 76). CD38 can also cleave NAD^+^ into its precursors, releasing nicotinamide mononucleotide and nicotinamide riboside (77). The enzyme can be found on the cell membrane with its catalytic domain either on the extracellular space or facing the cytoplasm, or on intracellular membranes (77). CD38 is a 300-aa long, type II membrane glycoprotein with a short cytoplasmic tail, a single transmembrane pass and the majority of the protein on the outer proportion of the plasma membrane (78, 79).

CD38 is considered a moonlighting enzyme because, aside its enzymatic function, it has been proposed to act as a co-receptor for various cell activation molecules. CD38 is expressed by naïve T cells, a subset of regulatory T cells, chronic infection-related T CD8^+^ cells, and thymocytes at the double positive stage (80, 81). When T lymphocytes are activated, CD38 synthesis increases, *via* Protein Kinase A and C (PKA PKC)-dependent pathway (82). Expression of CD38 in human B lymphocytes starts from early differentiation stages in the bone marrow, and it is maintained in mature B cells. In murine B lymphocytes CD38 expression is downregulated in cells entering germinal centers (83). Circulating monocytes express low levels of CD38, but its expression is increased by inflammatory stimuli and, interestingly, this is associated with differentiation into DCs (84). Other cells that express CD38 are NK cells, granulocytes and non-immune cells such as osteoclasts, erythrocytes, platelets, pancreatic and prostatic epithelial cells, neurons, astrocytes, muscle cells, renal tubular cells, retinal ganglion and cornea cells (85, 86).

Since CD38 has a short cytoplasmic tail, it was hypothesized that this molecule should interact with other membrane proteins to initiate intracellular signaling after activation upon antibody ligation. CD38 crosslinking in fact induces several cellular responses. Firstly, CD38 ligation on NK, T and B lymphocytes leads to proliferation and upregulation of activation markers (81). This molecule also participates during cell activation, potentiating signal transduction due to its close proximity to receptors mediating cell activation, such as the CD3/TCR complex in T cells, the surface Immunoglobulin (sIg), CD19, and CD81 in B cells, MHC class II in monocytes and CD16 in NK cells (77, 87). In naïve T cells and monocytes, CD38 crosslinking induces the secretion of both Th1 (IFNγ, M-CSF, IL-1β, IL-6) and Th2 cytokines (IL-4, IL-10 and IL-5) (73, 88). Activated neutrophils use CD38 to produce cyclic ADPR, which induces the release of intracellular Ca^2+^ that is required for responding to fMLP (75). In B lymphocytes, opposite consequences of CD38 activation have been reported: while its stimulation prevents apoptosis of B cells during clonal expansion in germinal centers, it also induces apoptosis of B cells precursors in the bone marrow, as well as of thymocytes in the thymus (80, 89, 90).

Regarding the role of lymphocytic CD38 in cell adhesion and migration, Dianzani, et al. first proposed that it mediates weak interactions between lymphocytes and the endothelium during the first steps of adhesion, before the stronger interactions occur (e.g. through integrins). They described two mechanisms for the participation of CD38 in adhesion: a direct interaction with an endothelial molecule, or by enabling the interaction of different lymphocytic membrane molecules with an endothelial ligand (91). A few years later, the same group reported that CD38 physically interacts with CD31 on endothelial cells and that this interaction leads to Ca^2+^ mobilization, which in turn leads to cytokine release, cell activation and cell-cell interaction with endothelial cells (92). Also, ligation induces CD38 molecules to aggregate in cholesterol and sphingolipids-rich membrane microdomains, which facilitates their internalization (93). It is plausible that such mechanism occurs during the first steps of adhesion, decelerating leukocytes in the bloodstream.

CD38–CD31 interactions are also established between human monocyte-derived dendritic cells (MDDCs) and endothelial cells. Migration induced by CCL-21 is inhibited either by blocking CD38 on MDDCs or CD31 on endothelial cells with specific antibodies (84). However, interaction with CD31 is not the only way that CD38 has for enabling cell migration; it was described that the reduced migration of DCs and neutrophils lacking CD38 is related to the lack of cADPR, which impairs Ca^2+^ mobilization (75, 92, 94). Thus, CD38 participates in migration in at least two ways: by promoting adhesion *via* CD31, and by the enzymatic production of cADPR, which induces Ca^2+^ mobilization required for migration.

CD38 ligation in T cells, either with monoclonal antibodies or with CD31, initiates a signal transduction cascade including the phosphorylation of PLC-γ, ZAP-70, MAPK and ERK2; whereas in monocytes the signaling pathway comprises PI3K and c-Cbl (73, 95, 96). This is noteworthy due to the fact that CD38 has only a short cytoplasmic tail with no known signaling motifs. Consequently, it is hypothesized that CD38 uses the signaling machinery of other molecules, such as the BCR, TCR, CD16 and MHC class II. The study of CD38 as a receptor started more than three decades ago and, as is true for several other cell membrane enzymes, more recently it has been refocused toward its expression and function in cancer cells, leading to the proposal of CD38 as a target for anti-cancer drugs (97–99).

## CD157

The ectoenzyme ADP-Ribosyl cyclase 2 (EC: 3.2.2.6), also known as CD157 or Bone Marrow Stromal Cell Antigen-1 (BST-1), is a glycosylphosphatidyl inositol-anchored glycoprotein with NAD glycol-hydrolase activity and ADP-ribosyl cyclase activity, like CD38, which belongs to the same gene family. They share 36% of their sequence and have similar pleiotropic functions both as receptors and as enzymes. Although CD38 has a transmembrane pass while CD157 does not, their structures are supported by 10 cysteines essential for disulphide-bond formation and enzymatic activity (78).

CD157 is expressed on myeloid cells, immature lymphocytes, bone marrow stromal cells, synoviocytes, endothelial and mesothelial cells, and dermal fibroblasts. Invasiveness of ovarian cancer epithelial cells is CD157-dependent, which increased expression promotes mesenchymal differentiation (100). The expression of CD157 is upregulated on neutrophils and basophils upon fMLP stimulation and on monocytes treated with MCP-1 (101–103), expression levels do not change on activated endothelial cells (104). CD157 expression on human umbilical vein endothelial cells (HUVEC) is restricted mainly to the intercellular region of membranes (104). CD157 plays a key role in transendothelial migration of neutrophils, as treating neutrophils with an anti-CD157 blocking antibody or treatment of HUVEC cells with an anti-CD31 blocking antibody, hinders their transendothelial migration (104). In humans, paroxysmal nocturnal hemoglobinuria is a genetic defect characterized by the attachment of glycolipids to proteins resulting in the absence of CD157 and other GPI-anchored proteins on the cell membrane, neutrophils from patients with this condition show impaired transmigration even though neutrophils adhere to the vessel wall (104).

CD157, as other GPI-anchored proteins, interacts with different transmembrane molecules to transduce signals. The antibody-induced clustering of CD157 on the cell membrane initiates a Ca^2+^-dependent cascade, F-actin reorganization toward the opposite pole of the cell, and β2 integrins activation in neutrophils (102). Moreover, CD157 co-localizes and functionally collaborates with β1 and β2 integrins for adhesion to Fibrinogen and Fibronectin (102, 103), inducing a signal transduction pathway that involves FAK, Src, AKT and ERK 1/2 (103). As long as β2 integrin CD18 is associated with CD11b to form MAC-1 (integrin αM/β2), CD157 co-immunoprecipitates with this heterodimer (105, 106), suggesting that CD157 can trigger intracellular signals using the MAC-1 (CD11b/CD18) signal transduction machinery.

Soluble recombinant CD157 binds to various ECM proteins such as Fibronectin, Fibrinogen, Collagen type I and Laminin, and these interactions can be inhibited by an anti-CD157 mAb or by heparin (107). Thus, CD157 participation in cellular adhesion of leukocytes may involve i) its association with integrins on the same cell membrane, ii) the interaction with components of the ECM and iii) direct interaction of CD157 with CD31 on endothelial cells. It is thus conceivable that CD157 interaction with CD31 is employed for transendothelial migration, while the interaction with Laminin, Collagen type I and Fibronectin could be involved in the cell´s migration through the tissues, enhancing the integrin-mediated adhesion to ECM. Finally, even though CD157 does not have signal transduction domains *per se*, its association with the CD18/CD11b heterodimer suggests that it may use the signal transduction cascade of this integrin.

## CD156

Human CD156, or ADAM-8 (A Disintegrin and Metalloproteinase domain-containing protein-8 (EC: 3.4.24.-)) is a type I transmembrane glycoprotein. ADAMs are conserved proteins that were initially related to the hemorrhagic snake venom protein (HSVP), which has a cysteine-rich region preceding the metalloproteinase domain. Structurally, ADAMs have a pro-domain that inhibits the active form of the enzyme, the catalytic domain, the disintegrin domain, a cysteine-rich region, a single transmembrane pass and a cytoplasmatic tail with signal transduction function (108). The intracellular tail of ADAM-8 has a proline-rich region similar to the SH3 (Src homology 3) binding sequence (109). ADAM-8 has a long list of substrates, including cell adhesion molecules, cytokine receptors and ECM proteins (110). ADAM family are metalloproteinases that bind to integrins through a RGD motif (Arg-Gly-Asp), inhibiting platelet aggregation (111).

Under physiological conditions, protein levels of ADAM-8 are low in monocytes, macrophages, granulocytes, dendritic cells, and endothelial cells, and even lower in B cells and neurons. But it has been reported that CD156 is upregulated by different inflammatory stimuli, such as Tumor Necrosis Factor-α (TNF-α) (112), Interleukins (IL) 4 and 13 (113), Lipopolysaccharide (LPS), Interferon-γ (IFN-γ), and by Peroxisome Proliferator-Activated Receptor-γ (PPAR-γ) (114, 115). ADAM-8 expression is also induced under inflammatory conditions in lung epithelium (113), osteoclasts (116), and glial cells in the central nervous system (CNS) (112).

ADAM-8 and ADAM-28 are the only members of the family that do not have a canonical sequence for Furin-like convertases between the pro-domain and the metalloproteinase domain (117). Thus, activation is achieved after their multimerization on the membrane and auto-catalytical cleavage of the pro-domain (117). The disintegrin domain has an integrin-binding loop with the residues KDM, followed by a cysteine-rich region and an EGF-like sequence. Interestingly, three different forms of ADAM-8 have been detected in cells: the inactive form with its pro- domain, the active form without the pro-domain, and the final form resulting from the removal of the metalloproteinase domain, leaving the disintegrin domain as the N-terminal domain (117). The first two forms are much less abundant than the last one. The remaining peptide (named DCE) consists of the disintegrin domain, and the cysteine-rich and EGF-like sequences; DCE participates in homophilic interactions with other cells (109, 117). Although the precise amino acids implicated have not been identified, the interactions with its ligands may include disulphide bonds, since reducing conditions impede adhesion (117).

CD156 molecules from both the endothelial cell and the leukocyte participate in the infiltration of myeloid cells into inflamed tissue. Migration of the monocytic human THP-1 cell line directed by CCL-2 was reduced when CD156 was downregulated using a shRNA. Similarly, reduced levels of CD156 diminished the migratory response of human neutrophils towards CXCL8. This effect on the migratory capacity was achieved either by chemically inhibiting CD156 enzymatic activity or by blocking the enzyme with monoclonal antibodies (115). Furthermore, expression of the α subunit of the integrin LFA-1 (integrin αL, CD11a) in monocytes is upregulated in response to CCL-2, but silencing of CD156 affected this upregulation, decreasing even more the adhesion of leukocytes to endothelial cells (115). These results were confirmed in an *in vivo* model where monocyte infiltration to inflamed lungs was less significant in ADAM-8^-/-^ than in wild type animals. Wound healing assays revealed that ADAM-8^-/-^ endothelial cells have significantly reduced migratory capacity (115). Although the mechanism is not yet completely understood, the evidence shows that migration is dependent on the enzymatic activity.

In order to accomplish cell adhesion, signal transduction through ADAM-8/CD156 is possible because unlike the other enzymes previously discussed, this enzyme has signaling motifs in its cytoplasmic tail. Potential candidates that could modulate the cascade are Src protein kinases (109), Cdc42-dependent actin assembly protein 1 (TOCA-1), and Cdc42-interacting protein 4 (CIP4) (110, 118). Additionally, ADAM-8/CD156 disintegrin domain interacts with β1 integrin inducing the FAK, ERK and protein kinase B (AKT) activation (110, 119). Precisely these pathways are implicated in ADAM-8-driven chemo-resistance and enhanced invasiveness of human glioblastoma cells (119). This suggests that ADAM-8 either modulates, or initiates signal transduction cascades and that it may be a potential therapeutic target in cancer.

## Conclusions

The hypothesis that ectoenzymes could transmit biochemical signals to the intracellular space was formulated before the term of moonlighting proteins was even coined (1). According to Stanley et al., all ectoenzymes must be integral proteins of the cell membrane with the catalytic site on the extracellular face of the membrane (1). However, ectoenzymes can also be found in other intracellular compartments, making larger the list of possible functions for each enzyme. Currently, several ectoenzymes are included in the category of moonlighting proteins, implying that these enzymes perform different functions depending on the cell type that expresses them, the timing, and the microenvironment, while they preserve their catalytic activity. Furthermore, in the past few years, a growing number of moonlighting enzymes expressed on immune cells have been shown to play a role in complex processes such as cellular adhesion, phagocytosis, and cell activation, among others.

**Figure 2:**
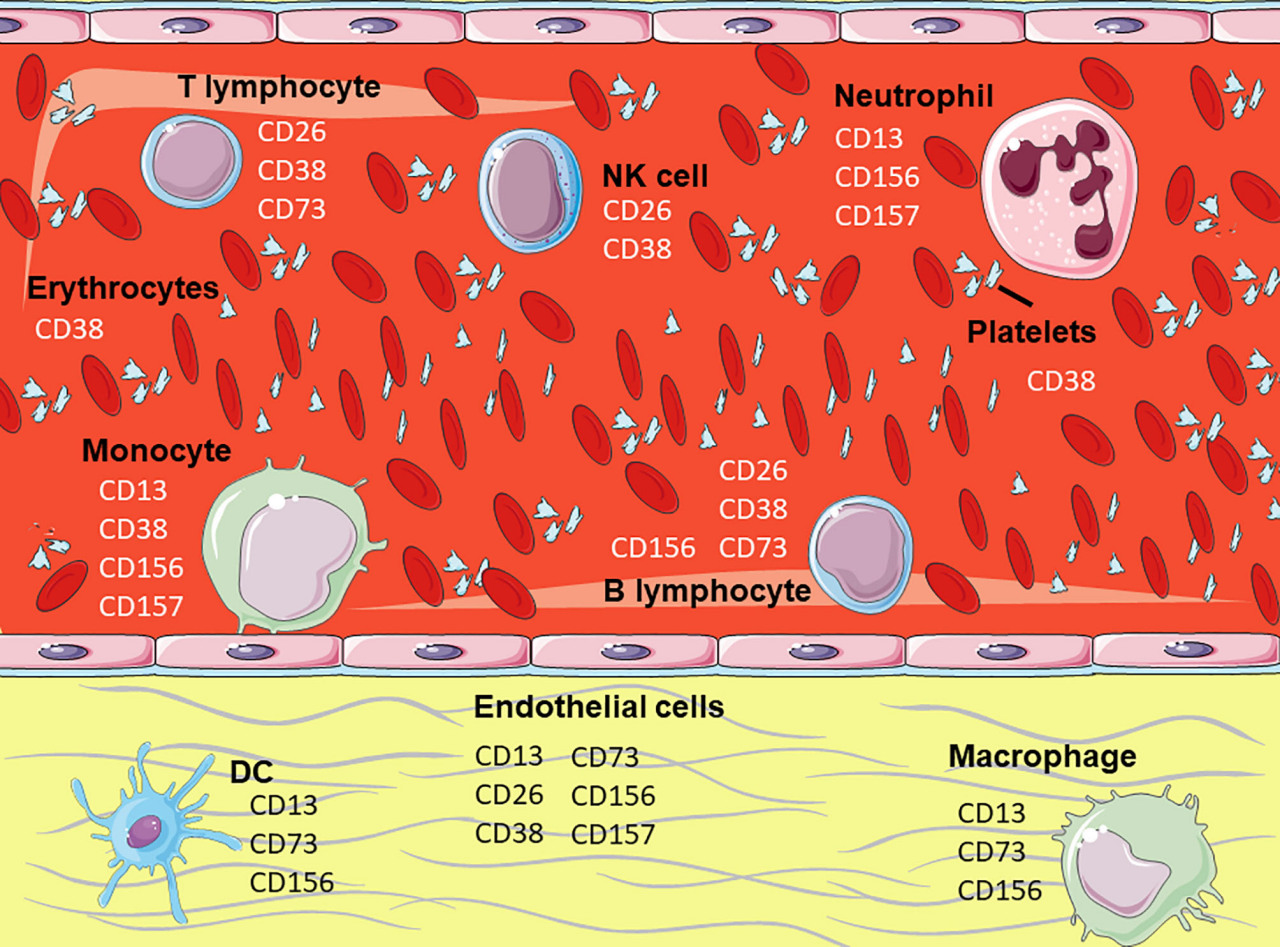
Expression of the membrane enzymes on hematopoietic cells. Representation of hematopoietic and endothelial cells highlighting the membrane enzymes involved in cell adhesion. Figure designed using images from Servier Medical Art (https://smart.servier.com/).

Ectoenzymes constitute a heterogeneous category of proteins that regulate multiple physiological processes through their different enzymatic properties, by cleaving peptides and hormones, processing molecules, clearing injured tissue, etc. (2). The products of their enzymatic activity trigger functional cellular responses that contribute to maintaining the homeostasis of the organism. The membrane enzymes discussed (**Figure 2**) contribute to the 4% of human leukocytes’ surface covered by ectoenzymes (7).

Each subpopulation of immune cells is endowed with a variety of specialized proteins that engage in processes such as phagocytosis, immunological synapses, antigen presentation, chemotaxis, and others. These add up to a variety of highly specialized mechanisms that maintain homeostasis, respond to danger signals, and scrutinize through the entire organism as sentinels. In certain cells, a number of enzymes have evolved to participate in cell adhesion. These ectoenzymes were probably selected not because they had conserved domains relevant for adhesion, but instead because certain chemical features of their structure enabled the cell to establish interactions with molecules on the surface of a different cell. Ultimately, chemical interactions and affinity to specific motifs are the initial motor for cell adhesion. This may have happened with some ectoenzymes on immune cells which suffered a positive selective pressure. In time, they became part of the cell´s array of molecules that mediate cell adhesion. However, the mechanisms by which these enzymes became capable of signal transduction should be part of a different evolutionary history. We hypothesize that the interaction of these enzymes with their ligands induced the aggregation of other adhesion molecules on the membrane that were included in the same microdomains (e.g. lipid rafts), and such molecules were originally responsible for signal transmission.

Since enzymes function in an orchestrated fashion, their expression and co-expression must be considered when formulating hypothesis on their evolution and physiology. For example in Jurkat and Raji cells, CD38 induces a dynamic mobilization of pre-synthesized cytoplasmic CD73 toward the membrane, where both function in the same axis (120). The biological significance of this synergism is still unclear; as both ectoenzymes share substrates, participate in lymphocyte activation and in the adhesion to the endothelium. In the case of CD73 and CD39, another nucleotidase, they take part side by side in the same ATP metabolic pathway, showing critical co-participation in the regulation of metabolism (55, 121). Interestingly, this cascade is used by several cancer cells and Treg cells to downregulate the immune response (55, 122). On the contrary, few cells co-express CD38 and CD157, which function similarly regarding their enzymatic and receptor activities (101).

In addition to ADAM-8, other ADAM proteins may be involved in cell adhesion. Distinct members of this family are overexpressed on cancer cells, and some of them have been shown to provide resistance to chemotherapeutic agents (123). Their deregulation during pathologies like cancer, reveal the importance of not only their catalytic activity, but also of the protein-protein interactions mediated by the disintegrin domain of ADAM proteins. For example, ADAM-12 and ADAM-15 bind to the α9β1 (124) and αvβ3 integrins (125); ADAM-9 to α6β1 integrin (126) and ADAM-23 to αvβ3 integrin (127). Although it is speculated that these interactions contribute to tumor growth and invasion, the fact that these same interactions are formed by ADAM molecules expressed on leukocytes awaits confirmation.

Cell adhesion is an ancient mechanism present since unicellular organisms, as it serves for cell communication. Thus, it is not surprising that many molecules have been evolutionarily selected to participate in such a complex process. Perhaps, selective pressures drove proteins toward the acquisition of post-translational modifications which made them part of this process. Finding other molecules that participate in cellular adhesion would help gaining a deeper understanding of the process *per se*. Also, as aforesaid, most of the molecules mentioned here are overexpressed in various cancers. However there are other disorders caused by adhesion molecules deregulated that could be treated more efficiently. This is the reason why many pharmacological developments attempt to target specific tissues with certain expression profiles (128). Furthermore, cell markers are especially useful for developing drugs, opening the door for more targeted and, hopefully, virtually side-effects free treatments.

## Author Contributions

GL-C: Conceptualization and writing the original draft. LD-A and EO manuscript critical revision. All authors contributed to the article and approved the submitted version.

## Funding

Work in the authors´ laboratory is funded by grants from CONACYT (Grant 87163) and PAPIIT-DGAPA-UNAM (IN 208320).

## Conflict of Interest

The authors declare that the research was conducted in the absence of any commercial or financial relationships that could be construed as a potential conflict of interest.

## Publisher’s Note

All claims expressed in this article are solely those of the authors and do not necessarily represent those of their affiliated organizations, or those of the publisher, the editors and the reviewers. Any product that may be evaluated in this article, or claim that may be made by its manufacturer, is not guaranteed or endorsed by the publisher.
